# Comparing thermal discomfort with skin temperature response of lower-limb prosthesis users during exercise

**DOI:** 10.1016/j.clinbiomech.2019.07.020

**Published:** 2019-10

**Authors:** Laura E. Diment, Mark S. Thompson, Jeroen H.M. Bergmann

**Affiliations:** Department of Engineering Science, University of Oxford, Parks Road, Oxford OX1 3PJ, UK

## Abstract

**Background:**

Thermal discomfort is prevalent among prosthesis users. This observational study of thirty unilateral lower-limb prosthesis users compared their skin temperatures and the thermal discomfort experienced during exercise between their residual and contralateral limbs.

**Methods:**

Participants performed a 2-minute interval cycling exercise test. Skin temperature was measured at matched locations on each leg during the 1-minute rest intervals. Average rate-of-change in skin temperature was compared between legs using a repeated measures analysis of variance. Participants rated thermal discomfort on each leg before and after exercise, and a Wilcoxon signed-rank test was used to compare legs. Ordinal regression evaluated the relationship between the rate-of-change in temperature on the residual limb and the perceived thermal discomfort.

**Findings:**

After exercise, thermal discomfort ranked higher on the amputated side (*P* = 0.007). On average, both legs cooled during exercise (*P* = 0.002), but the difference between legs was not significant. The rate-of change in skin temperature on the residual limb during exercise did not relate to the thermal discomfort experienced (odds ratio of 0.357).

**Interpretation:**

These findings indicate that in this patient population, skin temperature does not explain the thermal discomfort experienced, and subjective thermal discomfort is inadequate for detecting thermoregulatory issues, with potential implications for long-term tissue health.

## Highlights

• After exercise, thermal discomfort was substantially greater on the amputated leg.
• Skin temperature did not explain the thermal discomfort experienced.
• The skin on both legs cooled during intermittent exercise.
• The rate of temperature change differed between front and back of the leg.
• Above-knee amputees rated thermal discomfort higher than below-knee amputees did.

## 1 Introduction

Discomfort is a major concern identified by prosthesis users (Biddiss et al., 2007; Dillingham et al., 2001; Hagberg and Brånemark, 2001), with heat and perspiration in the prosthetic socket contributing substantially to the discomfort (Ghoseiri and Safari, 2014). Across 27 studies, an average of 54% of prosthesis users complained of heat-related issues (Ghoseiri and Safari, 2014), and thermal discomfort was the most prevalent type of discomfort experienced by prosthesis users in a tropical climate, with 71% experiencing thermal discomfort during daily activities (Diment et al., 2017). The insulating properties of conventional sockets and liners affect the amount of heat that dissipates through the socket and the body's temperature regulation (Klute and Rowe, 2007). Despite the prevalence of the issue, little is known about the way prostheses impact the body's thermoregulatory system and their contribution to thermal discomfort.

The skin is vital for thermoregulation (Romanovsky, 2014). Skin temperature is determined by the blood circulation to the skin, local metabolism, and heat exchange between the skin and the environment (Deng and Liu, 2004). As the body's internal temperatures increase, due to external factors or increased metabolism, the exposed skin uses three mechanisms to increase heat-loss to the environment so a healthy core temperature is maintained. These mechanisms are vasodilation of the blood vessels to increase blood flow through the surface capillaries, which increases heat loss through radiation, flattening of the hairs to remove the insulating layer of air around the skin to promote heat loss through convection, and sweat production by the sweat glands, which causes heat loss through evaporation.

The heat dissipation from exposed skin over active muscles during low intensity exercise is typically dominated by evaporation, causing the skin temperature to decrease (Neves et al., 2015). Wearing a prosthesis disrupts these cooling mechanisms and can cause skin temperature to rise, creating a warm, moist environment, prone to bacterial infection and skin breakdown (Dudek et al., 2005; Sanders et al., 1995). Heat and perspiration inside the socket can affect not only the tissue health, but also comfort, activity levels and prosthesis suspension (Ghoseiri and Safari, 2014). This build-up of heat on the skin during exercise was demonstrated in previous small studies by Klute et al. (Huff et al., 2008; Klute et al., 2014; Peery et al., 2005; Segal and Klute, 2016) and Cutti et al. (2014), who measured skin temperature within the transtibial prosthetic socket of amputees with traumatic etiology during rest, exercise and post-exercise recovery. These studies demonstrated that with low to moderate exercise, temperature in the socket increased.

No studies have compared the temperatures to matched locations on the contralateral limb, and only one assessed the thermal discomfort experienced, assessing cold discomfort when exercising in the snow (Segal and Klute, 2016). Additionally, the majority of leg amputations are attributed to vascular diseases (Ahmad et al., 2014; Stewart and Jain, 1992; Ziegler-Graham et al., 2008). This population is typically older and is at risk of low levels of total physical activity, minimal participation in sport, and high levels of sedentary behaviour. This means that they are unable to exercise for the lengths of time required in previous study protocols (Deans et al., 2012; Langford et al., 2019). Despite the sedentary behaviour, thermal discomfort remains an issue for this population (Ghoseiri and Safari, 2014). The other issue in this population is that vascular diseases can lead to peripheral neuropathy, where sensory loss can mean that the discomfort signals to the brain are disrupted, and tissue damage can occur without pain warning (Sorensen et al., 2006). It is therefore important to understand the mechanisms of heat transfer to prevent tissue damage.

This research focused on determining whether a subjective assessment of thermal discomfort is an adequate indicator of the body's thermoregulatory response to the prosthetic socket's thermal load. Individual resting temperatures vary substantially, and temperatures differ between the amputated and contralateral limb (Harden et al., 2008). Therefore, comparing the change in temperature between limbs, rather than assessing total temperature, enables a comparison of participants' responses to exercise and how well their bodies compensate for the increased thermal load of the prosthesis. By comparing the amount of change per minute, comparisons can be made across the population, even though some participants were not able to exercise for as long as others. Comparing rate-of-change in temperature also enabled a comparison between successive exercise intervals to assess whether there was a significant effect that might have been caused by adaption or fatigue in later exercise intervals.

The study aimed to assess: whether exercise caused greater thermal discomfort on the amputated limb in a prosthesis than on the contralateral limb; if the skin temperature response varied in rate-of-change between the residual limb in a prosthetic socket and the contralateral limb during exercise; and whether the rate-of-change of skin temperature on the residual limb related to the perceived thermal discomfort level.

Mobility India Rehabilitation Service Provision was chosen as the trial location because prosthetic discomfort and heat-related issues are prevalent in its tropical climate (Diment et al., 2017; Nagaraja et al., 2016), and it services an average of 350 prosthesis and orthosis users per month (Cochrane et al., 2015), enough to support the recruitment of 30 lower-limb prosthesis users for the study. Estimates on the number of amputees in India vary, but the 2011 census estimated that 5.4 million people had a mobility-related disability, including those with an amputation (Census of India 2011, 2011).

## 2 Methods

After receiving ethical approval through the Oxford Tropical Research Ethics Committee, 30 participants were recruited through Mobility India Rehabilitation Service Provision in Bangalore, between June and August 2016. All methods were carried out in accordance with relevant guidelines and regulations, and informed consent was obtained for all participants.

Participants met the following inclusion criteria: over 18 years old, had been a unilateral lower-limb amputee for at least 6 months, had a prosthesis that was in good working order, and were able to give informed consent to participating in the study. Participants were excluded if there was a potential risk of injury or medical complications when exercising for 6 min. Participants used their own prosthesis and had varying levels of amputation. All amputees who matched the inclusion criteria and either visited the Mobility India Rehabilitation Service Provision centre during the recruitment period, or were visited by the researcher and the community-based rehabilitation team, were invited to participate. The study took place at the rehabilitation service provision centre and in urban districts around Bangalore, where amputees involved in Mobility India's community-based rehabilitation program were located. A translator was provided by Mobility India in cases where the participant did not speak fluent English.

Each participant completed a survey with questions on demographics, the participant's amputation, the use of their prosthesis, and pain management. The demographics questionnaire was also provided in Kannada to help the translators.

**Fig. 1:**
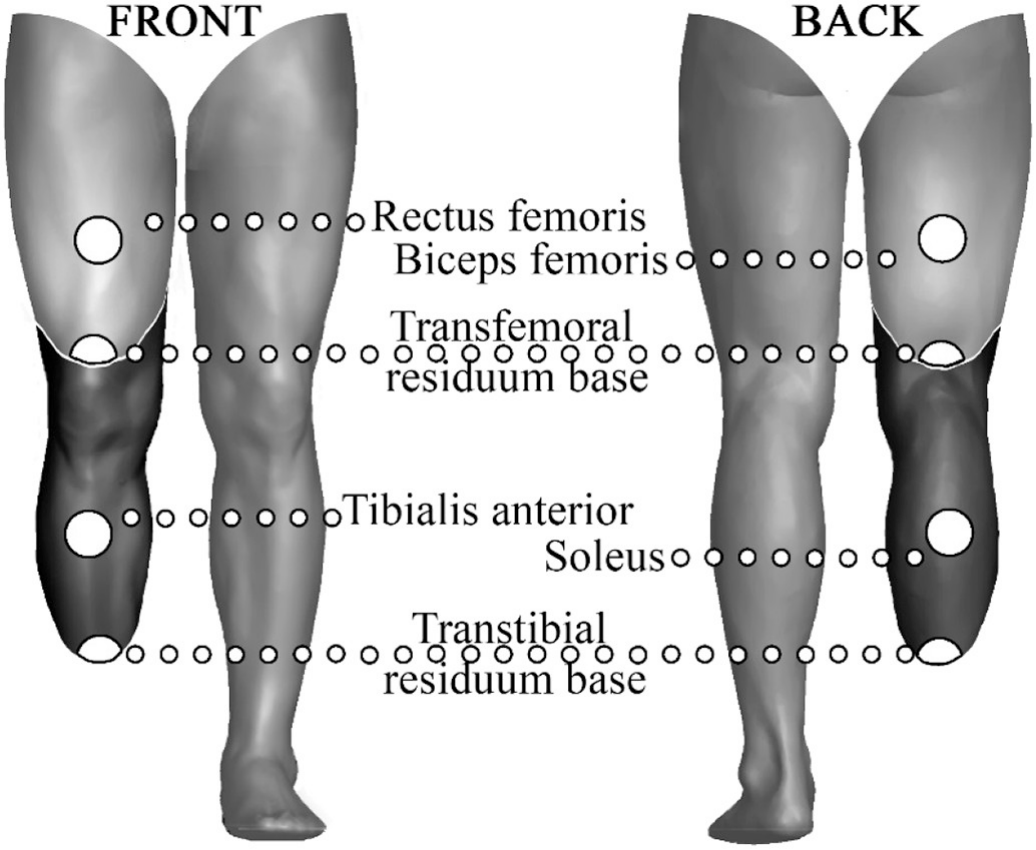
Temperature testing locations on the amputated limb.

The centre of muscle mass at the back of the thigh (for transfemoral amputees) or calf (for transtibial amputees) was marked with a pen, as well the same point at the front of the leg, equidistant from the base of the residual limb (Fig. 1). These locations were also marked on the contralateral limb, and used to collect measures of temperature throughout the study. The locations were chosen because muscle cells are the primary cells to generate and store heat during exercise (Despopoulos and Silbernagl, 2003), and therefore, measuring skin over the muscle is likely to give a good indicator of the temperature changes that occur during exercise.

Participants rested in a seated position for 10 min before the exercise test with their prosthesis on, so a baseline skin temperature recording could be taken at each location using a clinical infrared thermometer (Generation Guard, Victoria, Australia) with a 0.1 °C resolution. The prosthesis was removed for ~20 s to take the temperature readings, randomising the order of temperature readings between participants, and taking three readings at each location. Ambient humidity and temperature readings were also collected. To minimise variability in air velocity, all trials were performed in a room with no fans or air conditioning.

This study aimed to establish whether skin temperature can explain thermal discomfort experienced by amputees. Thus, the magnitude of the temperature response was less important than ensuring the socket fit was not disrupted by the sensors, and that the exercise was suitable for participants with limited fitness. A standard Submaximal Clinical Exercise Tolerance Test (SXTT) (Gappmaier, 2012) exercise protocol was used with a MagneTrainer (3D Innovations, Colorado, USA).The SXTT was chosen because it only required participants to exercise on the cycle ergometer in two-minute intervals with a workload suitable for their fitness level. A 1-minute rest was placed between each exercise interval, as described in Jones et al., which reduces issues with muscle fatigue in participants unaccustomed to cycling exercise, and made the exercise test accessible for prosthesis users with limited fitness (Jones et al., 1989). In this one-minute rest period, the temperature was recorded at each marker location, using the same protocol as the baseline temperature collection. The timing between doffing and taking the temperature readings was kept minimal and consistent across both legs and each time-point, with the order of readings randomised within and between participants. Muscles respond more slowly than skin to temperature changes (Miller et al., 2005), so if thermal discomfort increased without a significant change in skin temperature, it might signal that the heat generated by the muscles in the residual limb during exercise was being stored in internal tissues.

**Fig. 2:**
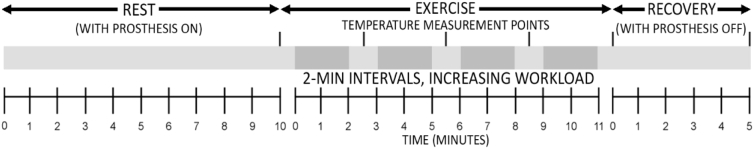
Exercise protocol timeline.

A self-administered specific activity questionnaire (McAuley et al., 2006) was completed prior to starting the exercise, to estimate the participant's metabolic equivalent score (MET). This was used to determine the incremental resistance to place on the pedals for each exercise interval. Music was played at 140 beats-per-minute during the exercise intervals, and participants were encouraged to pedal in time to the music to maintain a cycling frequency of 70 revolutions-per-minute (RPM). Assuming this pedalling rate, the protocols for increasing workload on the pedals were: MET scores of 1–4, 5–8, and > 9 started at 20 W, 20 W and 30 W, and increased by 5 W, 10 W and 15 W each exercise interval, respectively, as set out by Gappmaier (Gappmaier, 2012). The endpoint of the test was exhaustion, judged by inability to maintain a cycling frequency of 50 revolutions-per-minute, or when the participant asked to stop. After this final exercise interval, temperatures were taken at each location, then participants rested for 5 min with their prosthesis off before the final temperature recordings were taken. The protocol timeline is given in Fig. 2.

Immediately before and after the exercise test, participants were asked to rate their heat-related discomfort and discomfort caused by perspiration individually on each leg, using a 6-point scale from *no discomfort* to *intense discomfort*. Participants were also asked to complete historic discomfort scores retrospectively, using the same scale to get an indication of the maximum discomfort caused by heat and perspiration experienced over the previous month for each leg. A Wilcoxon signed-rank test was used to assess whether thermal discomfort differed between the amputated and contralateral limbs after exercise.

Lilliefors tests showed that each temperature dataset followed a normal distribution, and a two-way repeated measures analysis of variance (ANOVA) did not find a difference between the rates of temperature change of transfemoral and transtibial amputees (*P* > 0.05), so all participants were grouped together for analysis. A two-way repeated measures ANOVA was used to assess the temperature change over time to see whether there was a difference between the amputated and contralateral limbs and whether location of the temperature recording affected the response, with a *P*-value of <0.05 considered statistically significant.

Ordinal logistic regression was used to assess the relationship between the skin temperature changes that occurred on the residual limb during exercise and the perceived discomfort level, as well as to assess whether age, level of amputation or cause of amputation were associated with the discomfort experienced. The temperature of the residual limb at each time-point was calculated as the average of the 6 temperature readings taken (3 at the front and 3 at the back). The rate-of-change in temperature for each exercise interval was calculated as the average temperature of the participant's limb after the exercise interval minus the average temperature before the interval, divided by the 2-minute exercise period. Because participants exercised for different lengths of time, and the rate-of-change was not significantly different between exercise intervals, the average rate-of-change in temperature for each participant was measured as the average of their completed exercise intervals.

Multicollinearity between predictors was assessed by calculating the variance inflation factors to ensure these predictors were independent of one another. The prosthetic thermal discomfort score used to compare discomfort to skin temperature was based on removing each discomfort score taken on the contralateral limb from the matched discomfort score taken on the residual limb. To get a measure of thermal discomfort that differentiated between participants more than a single discomfort score, the perspiration discomfort score and past-month discomfort scores were included, with the thermal discomfort weighted twice as important as perspiration discomfort, and in-trial scores weighted twice as important as historic scores.

## 3 Results

There were 19 transtibial and 11 transfemoral amputees who participated, most of whom were male. There was large variation between participants in age range, years since amputation, and how long after the amputation each participant received their first prosthesis. The majority of amputees received a prosthesis within the first year, but some waited up to 13 years (Table 1).

**Table 1:** Responses to demographics questionnaire.

Topic	Factors (statistics given as: mean (median) (standard deviation))	Responses
Demographics	Age in years	44.4 (43.5) (SD 14.8)
	Gender (M/F)	25/5
	Level of amputation (femur/tibia)	11/19
	Years since amputation	8.8 (7) (SD 8.7)
	Months from amputation to first prosthesis	16.3 (9) (SD 28.2)
	Cause of amputation (trauma/disease)	19/11
Prosthesis use	Hours of prosthetic wear per day	10.2 (13) (SD 5.2)
	Training received (none/<1 day/<1 week/>1 week/forgotten)	7/4/4/14/1
	Activity level METs between 1 and 12	4.9 (4) (SD 2.2)
Education and occupation	Qualification level completed (none/primary/secondary/tertiary)	8/4/12/6
	Occupation before amputation (labourer/office worker/driver/home maker/not applicable)	16/5/2/2/5
	Self-stated reduction in occupation prospects post-amputation (yes/no/not applicable)	8/22/5
Pain management	Experienced pain today (yes/no)	11/19
	Uses medication for pain relief sometimes (yes/no)	5/25
	Used pain medication today (yes/no)	1/29

Most of the prosthetic sockets were fitted and manufactured at Mobility India, where they use anatomic suspension systems to focus the pressure on load-tolerant areas of the leg. The manufacturing materials used were resins, thermoplastics and laminates. Most participants wore a sock inside the socket, with 3 using multiple socks to improve comfort and fit. Two participants wore silicone liners, and one wore a copolymer liner.

Despite many participants stating that they often experienced pain or discomfort due to the prosthesis, and 11 experiencing pain in the 24 h leading up to the trial, most of the participants wore their prosthesis all day every day, and only 5 ever used pain relief medication (Table 1).

The ambient temperature was 26.5 °C (SD 1.3 °C), and the relative humidity was 72.8% (SD 5.4%). With the prosthesis on, the baseline temperatures of the residual limbs were not significantly different to the contralateral limbs.

No participants exercised for >8 min, and only 19 participants exercised for at least 6 min. Thirteen participants rated the residual limb as more uncomfortably hot after exercise than before, and 9 rated the residual limb as more uncomfortably hot than the contralateral limb after exercise. None of the participants rated the contralateral limb as more uncomfortably hot than the residual limb or felt that their amputated limb had cooled. The Wilcoxon signed-rank test showed that thermal discomfort was greater on the amputated side after exercise, with a mean score of 1.2/5, than on the contralateral side, which had a mean score of 0.6/5 (*Z* = −2.719, *P* = 0.007).

Despite participants stating that the amputated limb was more uncomfortably hot after exercise, the two-way repeated measures ANOVA showed that on average, the skin on both legs cooled during exercise (0.085 °C/min, *P* = 0.002). The difference between legs was not significant (*P* = 0.599). However, front and back measurements varied significantly in temperature response (*P* = 0.008), with the skin at the back significantly warmer than the front after exercise, in comparison to baseline.

**Fig. 3:**
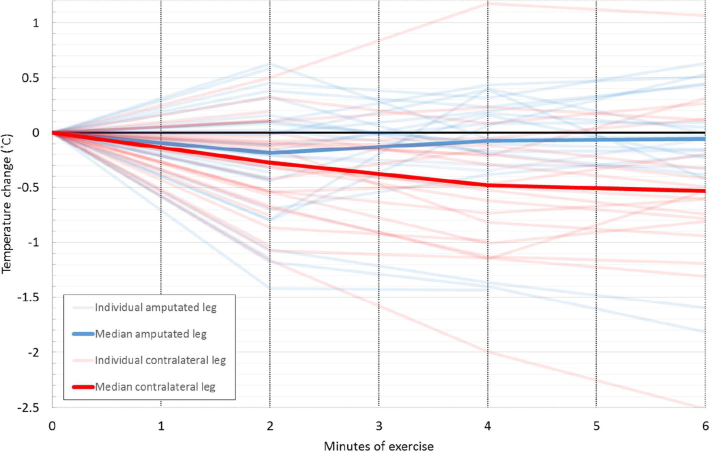
Skin temperature response during exercise.

The temperature responses during exercise for the amputated leg and the contralateral leg varied substantially between participants, with skin heating for some and cooling for others (Fig. 3). The temperature changes were small, with large standard deviations of approximately 0.5 °C on each limb. The average deviation between the 3 measurements taken at each location was 0.021 °C.

**Fig. 4:**
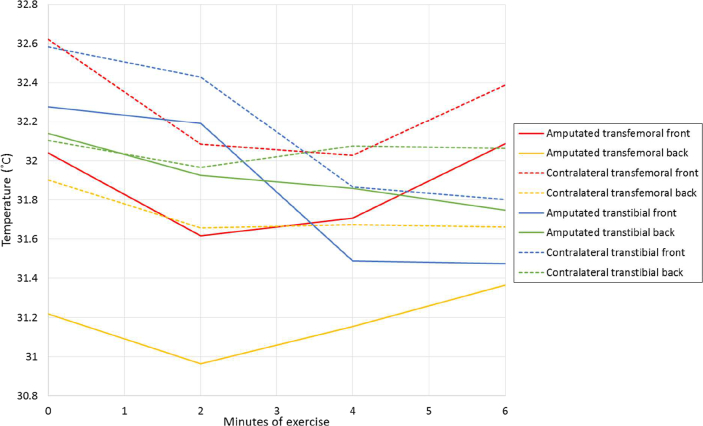
Differences in temperature between locations.

Fig. 4 shows the differences in temperature between measuring locations. The contralateral limb typically remained warmer than the amputated limb.

The raw data used in the ordinal logistic regression is provided in Table 2. The variance inflation factors for each independent variable compared to all others were small (<1.45), so multicollinearity between predictors was not an issue.

**Table 2:** Individual variables.

Participant	Thermal discomfort	Average rate of temperature change (residual limb °C/min)	Age (years)	Level (0 = transfemoral, 1 = transtibial)	Cause (0 = injury, 1 = disease)
1	2	−0.358	31	0	1
2	2.25	−0.050	61	0	1
3	0.75	−0.208	23	0	0
4	1.5	0.158	30	0	1
5	1.5	−0.303	55	0	0
6	2	−0.217	20	0	0
7	1.5	−0.023	42	0	1
8	0	0.073	45	0	1
9	3.5	−0.077	25	0	0
10	1	−0.004	41	0	0
11	1	−0.027	53	0	1
12	1.5	−0.117	35	1	0
13	4.25	−0.047	31	1	0
14	4.25	0.050	21	1	0
15	5.75	−0.114	48	1	0
16	2	−0.222	40	1	0
17	4.5	0.050	45	1	1
18	0.75	−0.053	65	1	0
19	2.25	−0.392	33	1	0
20	2.25	−0.125	30	1	0
21	1	−0.003	53	1	0
22	2.25	0.128	41	1	0
23	4	0.097	36	1	0
24	3.25	−0.183	65	1	1
25	0	−0.313	45	1	1
26	2	−0.258	62	1	1
27	0.5	0.433	59	1	1
28	3	−0.604	65	1	1
29	3.75	0.065	64	1	0
30	1.75	0.150	65	1	0

The temperature change on the residual limb with exercise was not associated with an increase in the odds of experiencing thermal discomfort (Table 3). The only statistically significant relationship with thermal discomfort was the level of amputation, which showed that transfemoral amputees rated thermal discomfort higher than transtibial amputees did.

**Table 3:** Relationship between thermal discomfort and various variables.

Variable	Odds ratio	Wald	95% confidence interval		Significance
			Lower bound	Upper bound	
Temperature	0.357	0.412	0.015	8.279	0.521
Age	0.969	1.468	0.920	1.020	0.226
Level of amputation	0.193	4.341	0.041	0.907	0.037
Cause of amputation	1.080	0.011	0.253	4.614	0.918

## 4 Discussion

After exercise, heat discomfort was experienced by participants in their residual limb, more so than in their contralateral limb. However, the skin temperature on both limbs decreased during the exercise. No difference was detected between the rate of decrease in temperature between the amputated and contralateral limbs. Neither was any association found between the temperature change on the amputated limb during exercise and the subjective rating of thermal discomfort. The fact that heat discomfort was experienced despite the skin temperatures typically decreasing, and the differences in temperature being smaller than average skin thermosensitivity (Stevens and Choo, 1998), suggests that the body's thermoregulation is adversely affected by the prosthesis, and that skin temperature is not a suitable predictor of thermoregulatory issues.

Thermal discomfort is typically used as a subjective “human sensor” to ensure action is taken when issues arise with thermoregulation, but the disconnect between the skin temperature responses and the thermal discomfort suggests that in this population the mechanism to detect thermal discomfort may be dysregulated on the amputated side, making it more difficult for the patient to correctly identify thermoregulatory issues and to adapt to the environmental constraints. This disruption to the system can lead to health issues related to a prolonged increase in core temperature, and may be partly due to peripheral neuropathy, which is particularly prevalent in diabetic amputees. It is therefore important to understand the thermoregulatory effects on the skin and in the muscles in order to create solutions that can counter thermoregulatory problems, rather than relying purely on a subjective measure of thermal discomfort. Though there was no difference between transfemoral and transtibial amputees in the average temperature change during exercise, transfemoral amputees rated thermal discomfort higher than transtibial amputees. This may suggest that losing more of the leg disrupts the body's ability to thermoregulate to a greater extent. Higher amputation levels have previously been associated with worse prosthetic outcomes and higher rates of prosthesis non-use (Biddiss and Chau, 2007).

Skin temperature responses vary according to the environment, and the exercise duration and intensity. Evaporation typically dominates heat dissipation from exposed skin over active muscles during low intensity aerobic exercise, causing the skin temperature to decrease, while high intensity anaerobic exercise typically causes the skin temperature over active muscles to increase (Neves et al., 2015). However, many factors influence the skin's capacity for heat loss and the subsequent skin temperature response, including ambient conditions, size, hydration, age, sex, fitness, acclimation, chronic health conditions, and clothing, including a prosthesis (Kenny and McGinn, 2017). The effect of these factors may explain why the skin temperature responses on both the amputated and contralateral limbs varied considerably from one participant to the next during exercise, despite using the same protocol in the same environmental conditions.

The prosthesis itself can disrupt the body's cooling mechanisms, as pressure on the pores limits surface blood vessel vasodilation, and the radiation and evaporation are significantly reduced by the insulating properties of the prosthetic socket (Klute and Rowe, 2007). Previous studies found that with longer periods of exercise without removing the prosthetic socket, temperatures in the socket increased (Cutti et al., 2014; Huff et al., 2008; Klute et al., 2014; Peery et al., 2005; Segal and Klute, 2016). This protocol instead recorded temperatures from intermittent low to moderate intensity exercise. Given that the majority of the amputee population are older adults and have limited fitness, this protocol may better represent the everyday physical exertion that leads to thermal discomfort.

It was expected that this intermittent exercise protocol with the prosthesis removed regularly would reduce the magnitude of the skin temperature response. However, the skin temperature response to a local environmental change, such as donning or doffing a prosthesis, is relatively slow (see supplementary material) (Fiala et al., 2001; Klute et al., 2014). Therefore a 1-minute rest to doff and don the prosthesis, leaving it off for ~20 s to take the temperature readings, was not expected to have a large, immediate influence on skin temperature. All readings were recorded digitally to reduce the data acquisition time and the time spent with the prosthesis off, keeping the recording time consistent across both legs and each time-point, with the order of readings randomised to reduce measuring error and bias. The study focused on the difference between the limb responses and the comparison with discomfort, rather than assessing the overall temperature change, to enable comparisons across the population despite individual resting temperatures varying substantially, and resting and doffing the prosthesis affecting the temperature response. The skin temperature in the socket decreased slightly on average over the exercise trial, suggesting that the removal of the socket between exercise intervals allowed some evaporative cooling to take place.

Using infrared thermometry to measure residual limb temperature differed from the previous studies, which used thermistors (Huff et al., 2008; Klute et al., 2014; Peery et al., 2005; Segal and Klute, 2016) or integrated circuit temperature sensors (ICs) (Cutti et al., 2014) taped on the skin in the prosthetic socket and wired to a data acquisition system. The reason infrared thermometry was used is because adding sensors inside the socket could affect the fit and comfort of the socket. Thermistors and ICs also do not directly measure the skin temperature, require calibration, are too expensive to be disposable and therefore require sterilisation, create self-heating errors that can affect the temperature output, and the accuracy is relatively low compared to the small temperature changes expected on the skin during short, intermittent exercise (MacRae et al., 2018).

Despite the skin cooling, thermal discomfort increased during exercise in the residual limb and not the contralateral limb. This suggests internal heating may be responsible for the thermal discomfort experienced. This theory is supported by the scientific literature that shows that limiting the body's ability to dissipate heat generated in the active muscles during exercise causes additional heat to be stored in the active muscles (Kenny and McGinn, 2017). Muscle temperatures respond almost instantaneously to changes in metabolism (Kenny et al., 2003), so they might associate more directly with thermal discomfort than skin temperatures. However, there is no easy way to measure muscle temperatures non-invasively.

The diverse individual variables; from cause of amputation, to age, size, sex, level of amputation, time since amputation, experience with a prosthesis, and prosthetic socket type, make comparisons across the population challenging. Therefore, because a computational model can assess the effect of independent variables and simulate internal temperatures, the development of a model to simulate the effect of the prosthesis on thermoregulation is recommended. Peery et al. developed a bioheat model to predict skin temperatures in a prosthetic socket at rest with no mechanical loading (Peery et al., 2006), but further work is required to investigate how physical activity with a prosthesis affects muscle and core temperatures. This may better diagnose thermoregulatory issues in this population than using a subjective “human sensor” such as thermal discomfort, and explain why thermal discomfort is experienced.

The small temperature changes during exercise, large standard deviations, and substantial difference in absolute temperatures between participants make it difficult to ascertain the clinical significance of these results. Many participants also did not maintain a consistent cycling frequency of 70 RPM, which could affect the individual skin temperature response. The fact that participants were unable or unwilling to exercise for >8 min may indicate that many of the prostheses were not adequate for exercise conditions. This theory is supported by the participants' self-evaluated MET scores, which gave a median of 4; associated with a fitness level that feels fatigue or shortness of breath if doing exercise as vigorous as sweeping floors, raking leaves, painting, or walking at a standard pace. All but two of the participants were unused to riding a bicycle, so the use of different muscles may have contributed to fatigue. However, a difference was not detected between the rates-of-change in temperature in successive exercise intervals, which suggests that the skin temperature was not substantially affected by fatigue or adaption to the exercise condition within this short exercise trial. Many participants were observed to have difficulties with donning and doffing their prosthesis, which also may have decreased their willingness to keep doing exercise intervals. A simulation enables longer, more intense exercise conditions to be evaluated than participants can manage with current prosthetics. It also allows a comparison of prosthetic materials and designs without the expense of manufacturing each. This approach can lead to a more data-driven method of designing future prosthetic sockets for individual thermal comfort.

Despite 11 participants experiencing residual limb pain in the previous 24 h, only 5 ever used pain relief medication for chronic residual limb pain and 24 wore their prosthesis for the majority of the day every day. This suggests that even though the prosthesis may have limited their ability to do physical exercise, most participants found significant benefit in having a prosthesis. The 5 participants who sometimes used pain relief medication used paracetamol, and one also used codeine. These both have temporary effects on body temperature and sensitivity to changes in temperature. However, no participants were using pain relief at the time of the trial. None of the other medications mentioned by participants have been linked to changes in thermoregulation or exercise performance.

The contralateral limb typically remained warmer than the amputated limb, despite the amputated limb being insulated by the prosthetic socket. This finding matches the scientific literature (Harden et al., 2008), and suggests a possible reduction in blood flow to the skin surface in the amputated limb that may be limiting the heat loss from the body, causing internal tissues to store additional heat.

Front and back of the leg were measured to improve the accuracy of the temperature reading for the limb. The rate of temperature change during exercise was different at the front of the socket to at the back, with the skin near the larger muscle mass at the back of the leg heating in comparison to the skin at the front. This supports the idea that the working muscles may be storing the heat. There were differences in temperature between measuring locations as well, which demonstrates the importance of considering anatomy in future analysis and in the development of a bioheat model. Developing a patient-specific model would enable the individual's factors that influence the body's capacity for heat loss to be considered, to assist in understanding the user's requirements for a prosthetic socket.

A standard numerical rating scale is widely used to measure pain, but has not been reliability tested for measuring discomfort caused by heat or perspiration, and the thermal discomfort score used in this paper, based on the heat and perspiration discomfort ratings, has not been validated. There is an inherent risk of bias when the participant is asked to think about heat and perspiration, as there is no way to blind the participant to the outcome assessment. This is another reason to develop an objective approach to assessing thermoregulatory issues, rather than using a subjective measure of heat discomfort.

## 5 Conclusions

These findings indicate that though heat discomfort was prevalent after exercising with a prosthesis, in this patient population, skin temperature does not explain the thermal discomfort experienced, and a subjective thermal discomfort score is inadequate for detecting thermoregulatory issues. Modern textiles that offer improved thermal regulation may be suitable for incorporating into future socket designs to reduce the thermal load on the body during physical activity (Gao et al., 2017), but little is yet known about the design requirements for a thermally comfortable prosthetic socket. A bioheat model has examined skin temperatures in a prosthetic socket during rest conditions (Peery et al., 2006), but further bioheat model development is recommended to increase understanding of the effects of a prosthesis on the body's thermoregulation and internal temperatures during exercise, and to enable a comparison of the thermal load on the body created by different socket designs.

## Appendix A Supplementary data

Able-bodied case-study to assess temperature of exercising with an insulating layer Image 1
