# Supplementary material for: Comparing thermal discomfort with skin temperature response of lower-limb prosthesis users during exercise
Source: Clin Biomech (Bristol). 2019 Oct;69:148–55. doi: 10.1016/j.clinbiomech.2019.07.020 (PMC6839397; doi:10.1016/j.clinbiomech.2019.07.020)
Supplement: Supplementary file 1 — Able-bodied case-study to assess temperature of exercising with an insulating layer [file mmc1.docx]

A case-study was performed with an able-bodied participant to assess the effect of exercising with an insulating layer and the effect of removing the insulating layer to take temperature measurements. The insulating layer was designed to have similar thermal properties to a prosthetic socket, and was worn on one leg. It consisted of a sock material as the inner lining, with a 2 mm thermoplastic sheet around it, moulded to the shape of the thigh from the epicondyles to the uppermost inner part of the thigh.


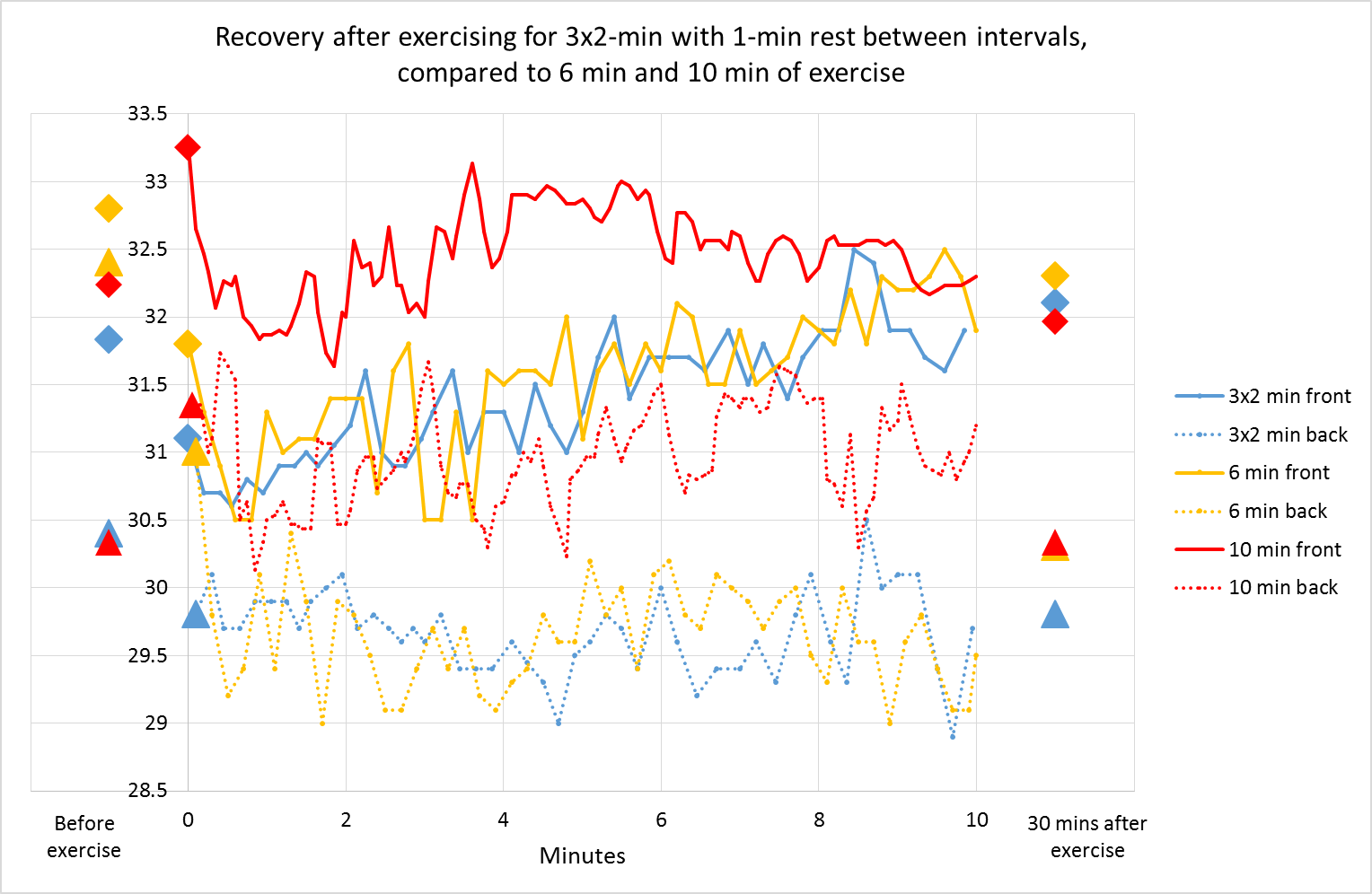


Figure 1. After exercising for different lengths of time with insulation around the thigh, the insulation was removed and the temperature change recorded


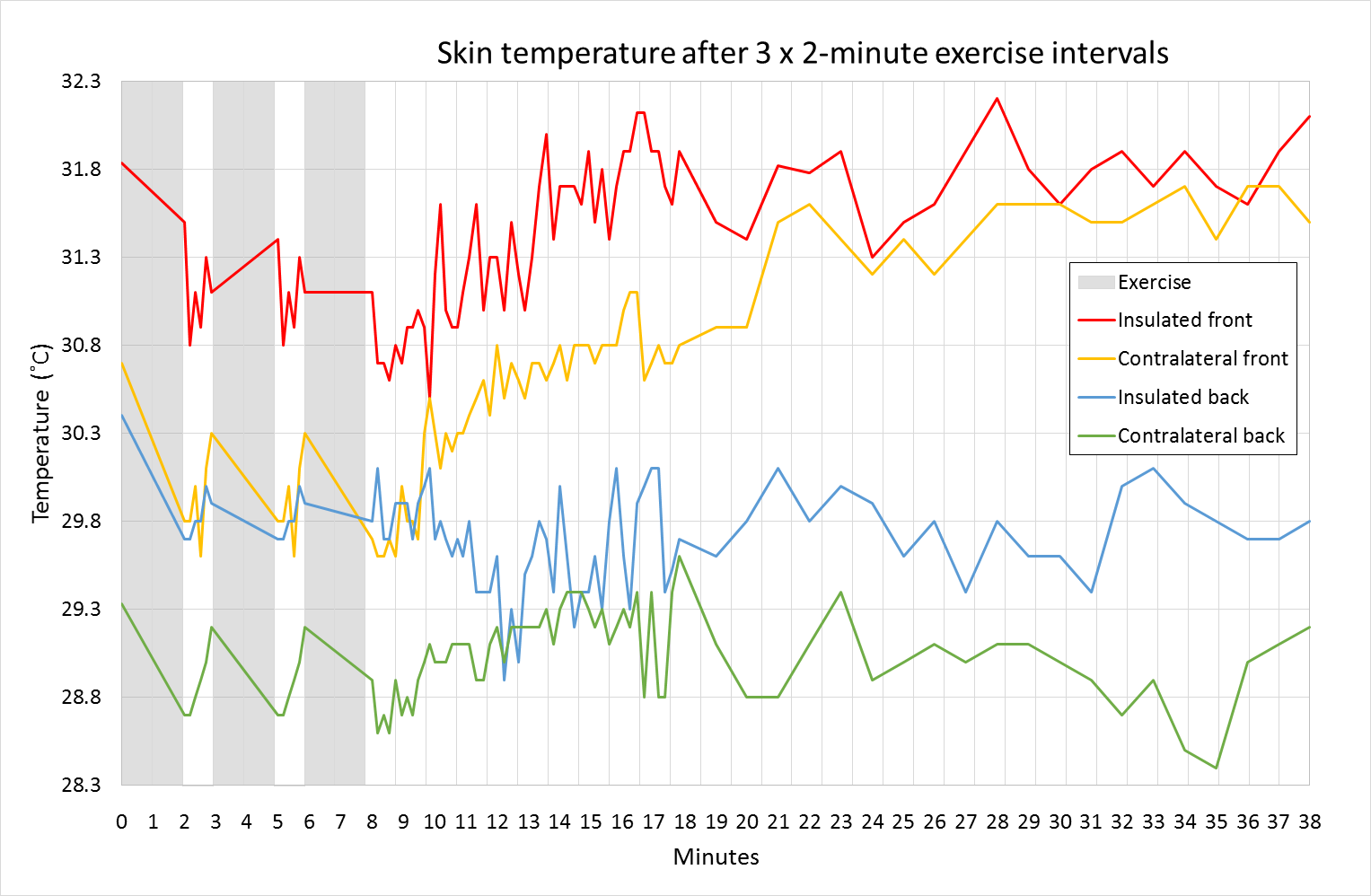


Figure 2. One thigh was insulated during the three 2-minute exercise intervals, and removed for a minute between each. This graph compares the temperatures between the insulated leg and the exposed leg.


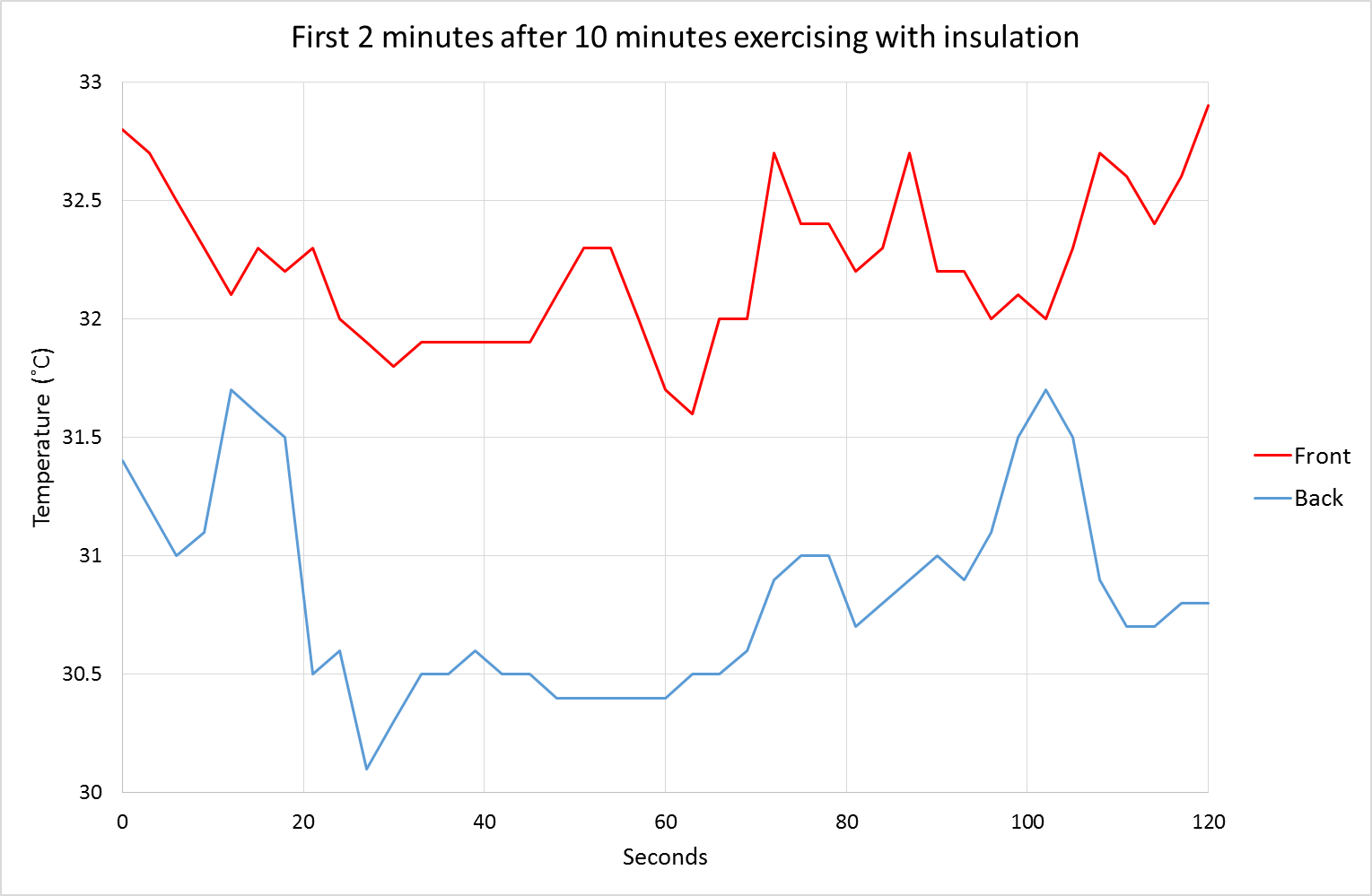


Figure 3. After exercising for 10 minutes with insulation applied to the thigh, the insulation was removed and the temperature recorded for 2 minutes
